# Perceived facilitators and barriers to self-management in individuals with traumatic spinal cord injury: a qualitative descriptive study

**DOI:** 10.1186/1471-2377-14-48

**Published:** 2014-03-13

**Authors:** Sarah EP Munce, Fiona Webster, Michael G Fehlings, Sharon E Straus, Eunice Jang, Susan B Jaglal

**Affiliations:** 1 Institute of Health, Policy, Management and Evaluation, University of Toronto, Toronto, Canada; 2 Department of Family and Community Medicine, University of Toronto, Toronto, Canada; 3 Division of Neurosurgery, Department of Surgery, University of Toronto, Toronto, Ontario, Canada; 4 Li Ka Shing Knowledge Institute of St. Michael’s Hospital, Toronto, Ontario, Canada; 5 Department of Applied Psychology & Human Development, Ontario Institute for Studies in Education, Toronto, Ontario, Canada; 6 Department of Physical Therapy, University of Toronto, Toronto, Canada; 7 Toronto Rehabilitation Institute, Toronto, Canada

**Keywords:** Facilitators, Barriers, Self-management, Traumatic, Spinal cord injury, Qualitative

## Abstract

**Background:**

Current evidence has suggested the need for increased self-management support efforts in spinal cord injury (SCI) to reduce secondary complications. However, current self-management programs may not be suitable for the unique needs of individuals with SCI, including reduced mobility and the importance of attendant care. There is a need for greater understanding of the self-management strategies adopted by individuals with SCI and the potential need for a tailored self-management program. Thus, the purpose of the current study was to understand the perceived facilitators and barriers to self-management to prevent secondary complications.

**Methods:**

A descriptive qualitative approach was used and involved telephone interviews. Semi-structured interviews were conducted with individuals with traumatic SCI, their family members/caregivers, and managers from acute care/trauma and rehabilitation centres. Participants were recruited between September 2011 and May 2012. Analysis was conducted using inductive thematic analysis to understand the perceived facilitators and barriers to self-management to prevent secondary complications.

**Results:**

A total of 26 interviews were conducted and they included 7 individuals with traumatic SCI, 7 family/caregivers (i.e., 7 SCI-caregiver dyads), and 12 acute care/rehabilitation managers from across the province of Ontario. The following five facilitators to self-management were identified: physical support from the caregiver, emotional support from the caregiver, peer support and feedback, importance of positive outlook and acceptance, and maintaining independence/control over care. The following five barriers to self-management were identified: caregiver burnout, funding and funding policies, lack of accessibility, physical limitations and secondary complications, and difficulties achieving positive outlook or mood.

**Conclusions:**

This study demonstrated that the caregiver and the individual’s own mood/outlook, among other facilitators and barriers, make significant contributions to the self-management of individuals with traumatic SCI. The issues of timing/readiness and comorbidities and aging were observed across many of these themes. As such, the development of a tailored self-management program for individuals with traumatic SCI and their caregivers should incorporate these considerations.

## Background

### Spinal cord injury and secondary complications and health care utilization

A spinal cord injury (SCI) results in a number of motor, sensory, and autonomic impairments which predisposes a person to multisystem dysfunction, leading to an increased likelihood of a range of related secondary complications [1-4], defined as medical consequences that can cause functional limitations [5]. A cross-sectional study from the US Model System determined that 95.6% of patients had at least one medical complication at the time of their routine annual check-up [6]. Common secondary health complications after SCI include pressure ulcers, urinary tract infections, bowel problems, fractures, chronic pain, and depressive disorders [5]. Despite the fact that many of these complications are amenable to treatment and/or prevention, secondary complications represent a significant burden at both the health system and individual level: they are costly, in terms of limited health-care resources [7-9], and they intensify the experience of disability for people with SCI by negatively impacting on long-term health, productivity/employment, dignity, mobility, and independence [10].

As a result of secondary complications, individuals with SCI have greater rates of contact with the health care system than the general population, and also have multiple rehospitalizations throughout their lifetime. Jaglal and colleagues [11] reported a one year readmission rate of 27.5% among individuals with traumatic SCI in Ontario. Secondary complications were the main reasons for readmission. In a related study, a high number of visits to family physicians and physiatrists were reported [12]. The authors concluded that the high rate of physician and specialist utilization, emergency department visits, and hospital readmissions, in and of themselves, indicate that current self-care practices are not managing or preventing secondary complications adequately and indicated that future research should examine preventive strategies that could be implemented in order to improve the long-term quality and cost of care for persons with traumatic SCI [11,12].

### Need for preventative strategies in the community

Current evidence has suggested the need for increased SCI self-management efforts to reduce secondary complications, including knowledge of risk and protective factors, skills to minimize risk, social support, and timely referral to professional health care [13]. A study of care received, care needs and preventability of secondary complications among persons with long-term SCI living at home reported that there were substantial unmet needs [14]. Information, psychosocial care, and self-efficacy were areas that needed to be enhanced and the need to explore self-management strategies was recommended. Similarly, Pang and colleagues [15] determined that confidence or self-efficacy to manage SCI in many community-living people with SCI is suboptimal.

### Chronic disease self-management program in spinal cord injury

A qualitative study on the experiences of individuals with neurological conditions, including stroke, multiple sclerosis, and SCI, who participated in the Stanford Chronic Disease Self-Management Program, determined that the participants with SCI reported the least satisfaction with the program [16]. Individuals with SCI as well as some of the facilitators themselves suggested assembling a SCI-focused group (e.g., individuals with SCI needed information specific to and modules adopted for being in a wheelchair/reduced mobility). In addition, they also found that when attendant care is an important component (as is the case in individuals with SCI), a different approach may be needed to teach self-management skills (i.e., being a good director of care, instead of a person who actually manages care independently) [16]. Collectively, these findings point to the need for greater understanding of the self-management strategies adopted by individuals with SCI who are managing well. This information could then be used to inform the components of a self-management program for individuals with SCI. For the purposes of this study, self-management is defined as: “…*the tasks that individuals must undertake to live with one or more chronic conditions. These tasks include having the confidence to deal with the medical management, role management, and emotional management of their conditions*” [17].

### Research objectives

The objective of the current study was to understand the perceived facilitators and barriers to self-management to prevent secondary complications from the perspectives of individuals with traumatic SCI, their family members/caregivers, and managers from acute care/trauma and rehabilitation centres. This is the first study to the best of our knowledge to understand the facilitators and barriers to self-management in SCI and from such multiple perspectives.

## Methods

### Conceptual framework

Key processes of the Knowledge to Action framework informed the current research initiative [18]. This framework incorporates the common elements of more than 30 planned action theories and was developed by Graham and colleagues [18]. It has been adopted by the Canadian Institutes of Health Research as the accepted model for promoting the application of research and a framework for the process of knowledge translation. The current study focuses on the facilitators and barriers phase with the view to developing a new self-management program based on the facilitators and barriers identified in the current study.

### Design/approach

The present study took a descriptive qualitative approach using telephone interviews. This approach was employed as there is a paucity of research on self-management in individuals with traumatic SCI as well as their caregivers, and the qualitative descriptive approach is well-accepted for researching topics about which little is known and yielding practical answers of relevance to policy makers and health care practitioners [19,20]. Given the potentially important role that caregivers have in the self-management of individuals with SCI, as outlined above, individuals with traumatic SCI and their caregivers (“the SCI-caregiver dyad”) were included. Health care (or clinical) managers from adult acute care/trauma and rehabilitation centres were included in order to triangulate the findings from a health care professional and/or health system perspective (i.e., presumably, the managers would have both clinical and health system knowledge related to individuals with SCI and their families). Given the geographic diversity as well as the potential accessibility limitations of the study participants, telephone interviews were conducted. Using this approach, it is assumed that the current findings could be used to develop a tailored self-management program for individuals with traumatic SCI. Research ethics approval was obtained from the University of Toronto (Protocol Reference #26429). All participants provided informed consent prior to the interview.

### Recruitment

Community-based (i.e., non-hospital based) individuals with traumatic SCI were recruited via 1) an online advertisement posted on the SCI Canada-Ontario web site; 2) a print advertisement included in the SCI Canada-Ontario magazine “Outspoken”; 3) postings and direct personal interactions with Regional Services Coordinators from various SCI Canada-Ontario branches; and, 4) a community exercise rehabilitation program at McMaster University in Hamilton, Ontario (“MacWheelers”). Purposive sampling was used to identify and subsequently recruit study participants [21]. Some of the criteria for purposeful sampling included participants’ urban and rural status. Individuals with traumatic SCI who were interested in the study contacted the principal investigator (SM) by telephone or email to inquire about the study. Eligible participants included individuals who were 1) 18 years of age or older; 2) fluent in English; 3) had experienced a traumatic SCI (e.g., a fall, motor vehicle accident, sporting accident, etc.); and, 4) who had a formal or informal caregiver who was willing to participate. Caregivers/family members were recruited via the individuals with traumatic SCI and were identified as the individual’s primary caregiver. Individuals with traumatic SCI and their family member/caregiver were interviewed separately to mitigate potential power imbalances, which would influence the experiences they would be willing to share. The contact information of managers from acute care/trauma and rehabilitation centres across Ontario that are recognized for treating individuals with SCI was identified via Internet searches. Managers were subsequently contacted by telephone, informed of the study, and asked whether or not they wished to be interviewed. Participants were recruited between September 2011 and May 2012. Recruitment ceased as the study approached the point of data saturation, which is the point when successive interviews become repetitive and no new responses or themes emerged [22].

### Data collection

Each participant took part in a semi-structured telephone interview lasting approximately 60–75 minutes. The interviews were conducted by the principal investigator (SM). The interview guide consisted of semi-structured open-ended questions (see List of Examples of Open-Ended Questions from Interview Guide) and was pilot tested with a scientist experienced in qualitative methods (FW) as well as an individual with a SCI. Probes or recursive questioning were used during interviews to explore issues in greater depth and verify the interviewer’s understanding of the information being collected [21]. Slight variations existed in the interview guide depending on the participant group (see Table 1). The complete list of questions is included in Additional file 1. All interviews were digitally recorded and transcribed verbatim for data analysis.

**Table 1:** Characteristics of individuals with traumatic spinal cord injury

**Characteristic**	**N = 7; n, Range**
**Sex**	
Male	6
Female	1
**Age**	39-68
**Time since injury (years)**	2-25
**Level of injury**	
Paraplegia	5
Quadriplegia	2
**Education**	
<High School	2
Undergraduate/college	4
Post-graduate	1
**Employment status**	
Unemployed/retired	5
Part-time	1
Full-time	1

List of examples of open-ended questions from Interview Guide:

1a. What are some of the factors that have contributed to success in self-management?

1b. Manager version: What do you believe are the facilitators to self-management for patients in the community at the individual, provider, and/or policy levels?

2a. What are some of the factors that have impeded success in*/ability to maintain* self-management?

2b. Manager version: What do you believe are the barriers to self-management for patients in the community at the individual, provider, and/or policy levels?

2c. Manager version: What could be added to your program to assist patients with self-management support/skills? What are the facilitators, barriers to making this/these addition(s) at the individual, provider, and/or policy levels?

3. What are you currently doing to prevent any secondary complications**,** that is, any medical conditions that arise as a result of your spinal cord injury, such as urinary tract infections or pressure ulcers?

4. What prompted your last visit to hospital or your physician’s office? What kind of *specific* help did you need after that visit *(attendant care, etc*.)? Who or what helped you meet these needs? Was there anything that was not helpful? Example of Probes: How so? Tell me more about that.

### Data analysis

Data collection and analysis were carried out in an iterative manner. The accuracy of the transcripts was verified by the interviewer (SM). All identifying information was then removed from the transcripts prior to being reviewed by the other team members. Analysis was conducted using inductive thematic analysis as described by Braun and Clark [23] in order to understand the perceived facilitators and barriers to self-management in traumatic SCI. A subset of the interview transcripts were initially coded by the principal investigator, giving full attention to all data. In addition, two other researchers (SJ, SM) independently coded this same subset and met to compare their codes. This step allowed for enhanced reflexivity and ensured rigour. A coding framework was developed and applied by the principal investigator to the remaining transcripts. To facilitate the organization and analysis of the qualitative data, the principal investigator’s reflective notes from the interviews, as well as the transcripts were entered into NVivo 9 [24]. Following this, the codes were clustered into groups or categories (i.e., codes that shared similar meanings) and predominant themes were identified. To maximize credibility and trustworthiness, three members of the research team (SM, SJ, FW) then met over several meetings to discuss the developing analysis. New themes were also discussed. Together, the researchers explored various thematic maps until consensus was reached and theme labels were agreed. The principal investigator analyzed the remaining data.

## Results

### Description of participants

A total of 26 interviews were conducted, which included 7 individuals with traumatic SCI and 7 family/caregivers (i.e., 7 dyads), and 12 acute care/rehabilitation managers from across the province. Characteristics of the individuals with traumatic SCI are reported in Table 1. In terms of the family member/caregiver group, five were spouses (female), one was a sibling (male), and one was a personal support worker (female). The age range of the family members/caregivers was 39 to 65 years of age. All of the acute care/trauma and rehabilitation managers were female with an age range of 36 to 62 years of age. The number of beds at the centres that these managers represented ranged from 12 to 63. Overall, 7 of the 26 participants lived in Northern Ontario. To protect anonymity, quotes exemplifying the various themes only include the participant’s group (i.e., individuals with traumatic SCI, family member/caregiver, manager) and his or her sex.

### Facilitators to self-management

The following five facilitators to self-management were identified: physical caregiver support, emotional caregiver support, peer support and feedback, importance of positive outlook and acceptance, and maintaining independence/control over care. Representative quotes of the facilitators to self-management have been compiled in Table 2.

**Table 2:** Themes and representative quotes of facilitators to self-management

**Theme: Physical support from the caregiver**	
**Representative quote**	**Source**
*“I do all the cooking, the cleaning. I was doing snow removal but then I gave it up. I do the grass cutting. I mean if there are light bulbs to be changed, just general maintenance around the house, anything that he can’t manage and the grocery shopping and the bill paying”*	Caregiver 6; Wife of individual with traumatic SCI
*“I mean some people will never be able to self-catheterize. So we educate their partner in care as to how they can help to do that. So they need to be taught at the same time as the individual patient. They need to know the risks in particular with you know I’m thinking of bladder dystonia and pressure sores, transferring and all of that. I mean these people aren’t going home to live by themselves. That’s quite rare. So they need to have the support service from their partner in care and family members and they need as much education as the patient does, sometimes more”*	Manager 4; Female Rehabilitation Manager
**Theme: Emotional support from the caregiver**	
**Representative quote**	**Source**
*“When I’m confronted with situations like that, I kind of sort of buckle and break down. Thank God I have a husband who’s like a really strong advocate because I’d say he’s really more of the advocate than I am and when things go bad, he’s the one that can step in and advocate on my behalf to make sure that I get what I need”*	TSCI 1; Female with traumatic SCI
**Theme: Peer support and feedback**	
**Representative quote**	**Source**
*“Often times when the patient is here or even when they’re in acute care, the CPA will hook up…and begin that dialogue about getting back into the community. While they’re inpatients here they also have what they call peer support workers. So they will buddy someone who’s had a former injury with a patient here who might have the same level of injury, be close in age. I think those are a couple of excellent facilitators for patients”*	Manager 11; Female Rehabilitation Manager
**Theme: Positive outlook and acceptance**	
**Representative quote**	**Source**
*“I accept the fact that…it was very difficult but I finally did accept the fact that I’m a quadriplegic and I’m going to be like this for the rest of my life. So I may as well just accept it and get on with it”*	TSCI 2; Male with traumatic SCI
*“With J., he has had jobs where he’s working, he has to get up…so he has to be at certain things. So he has to get out of bed. He has things to do. I could definitely… that would be something with other people in a wheelchair, there wouldn’t be any motivation and that affects everything; self-esteem, social skills. Then of course it affects their health because they’re not moving, they’re not doing things. They don’t have to be somewhere”*	Caregiver 4; Wife of individual with traumatic SCI
**Theme: Maintaining independence/control over care**	
**Representative quote**	**Source**
*“Well the idea of…for instance I knew that I needed some sort of satisfaction happy environment. So I decided right away to go back to drive a car. I remember I became very eager at that and I said that’s it, I could have a car”*	TSCI 7; Male with traumatic SCI

#### Physical support from the caregiver

Participants across the three groups noted the significant role that caregivers played in terms of providing physical support. Physical support was described by participants as assistance with basic (e.g., bathing, dressing) and instrumental (e.g., housework, meal preparation) activities of daily living as well as assistance with the prevention/monitoring and/or management of secondary complications. It was noted that some of these supportive skills (e.g., bowel and bladder management, wound care) were taught to family members early in the course of the patient’s rehabilitation stay and staff recognized the importance of family members in this role. There was also recognition of aging caregivers and/or caregivers with one or more chronic condition(s) themselves. As a result, participants expressed concern about the sustainability of this support and even the potential absence of this support.

#### Emotional support from the caregiver

Similarly, the significant role of the caregiver in terms of providing emotional support to the individual with the traumatic SCI was recognized, particularly among the individuals with the traumatic SCI as well as their caregivers. This type of support often involved encouraging and advocating for the individual with SCI.

#### Peer support and feedback

Peer support and feedback was also described as an important facilitator to self-management. Most participants referenced the Peer Support Program provided by SCI Canada and highlighted its value in pairing a newly injured individual with an individual who is several years post-injury. During peer support interactions, it was noted that the newly injured individual could share his or her fears/frustrations and that the individual who was several years post-injury could share his or her own experiences and thus offer assurances and/or expectations in the recovery course. The timing of this support was noted by several participants (e.g., that the individual with the traumatic SCI may not be ready to receive the support and/or information) as well as the need for considering and even matching some of the characteristics of the two parties (e.g., age, sex, level of injury). Some participants noted matching is an existing practice of SCI Canada when possible.

#### Importance of positive outlook and acceptance

In addition to the beneficial roles of both family members/caregivers and peers, as described above, the individual’s own positive outlook and acceptance was stressed across the participant groups as being key to their effective self-management. Some participants described this positive outlook as a characteristic that existed before the injury, but at the same time, it was also recognized that an individual’s outlook could evolve over the course of recovery. Finally, the potential roles of working or volunteering were recognized as contributing factors to this positive outlook.

#### Maintaining independence/control over care

Across all participant groups, the importance of the individual with SCI maintaining control over care also emerged as an important facilitator to self-management. In some instances, this independence was tied to increased mobility, and specifically, having access to a vehicle and being able to drive.

### Barriers to self-management

**Figure 1:**
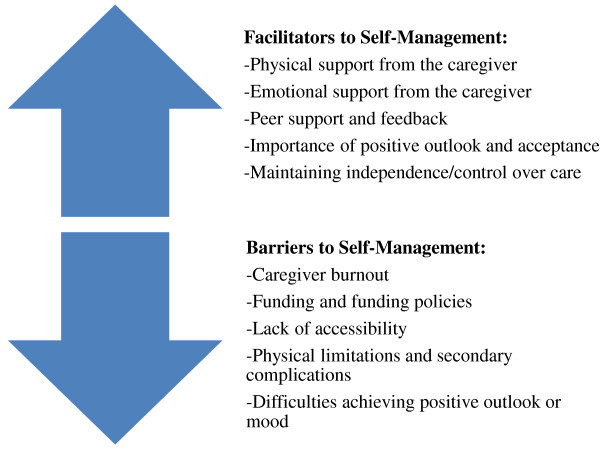
Perceived Facilitators and Barriers to Self-Management across Individuals with Traumatic Spinal Cord Injury, their Family Members/Caregivers, and Acute Care/Trauma and Rehabilitation Managers.

The following five barriers to self-management were identified: caregiver burnout, funding and funding policies, lack of accessibility, physical limitations/secondary complications, and difficulties achieving positive outlook or mood. Representative quotes of the barriers to self-management have been compiled in Table 3. The facilitators and barriers have also been represented in Figure 1.

**Table 3:** Themes and representative quotes of barriers to self-management

**Theme: Caregiver burnout**	
**Representative quote**	**Source**
*“My concern is you get people who, especially if it’s a husband and wife thing where they don’t have the insurance and the husband has gotten to the point where I don’t want to do this anymore for myself and then the wife has to do it. That changes the dynamics too much. It has to happen regularly. How can you… I don’t know. How can you provide a hug and a kiss to somebody where you’ve just done their bowel care? It’s just too hard you know?”*	Manager 2; Female Rehabilitation Manager
*“So in pediatrics they have a family support service. Here [in the adult rehabilitation system] there’s not that same support. I’ve never even heard of a family support service. That helps them to understand even emotionally where they’re at so that are they able to be dealing with this…”*	Manager 12; Female Rehabilitation Manager
**Theme: Funding and funding policies**	
**Representative quote**	**Source**
*“You know before we had a whole bunch of cuts happen, we used to have OTs [occupational therapists] go around. They would do certain rounds in the morning with the patients, the ones who have modified hand function. They would go around and take a look at and be there with the patient while they’re getting dressed and give the patient tips and see if they need adaptive devices that help them to put their socks on and get dressed and put their shoes on and also brushing their teeth”.*	Manager 2; Female Rehabilitation Manager
*“Yes, it’s only auto insurance that provides case management. If you had a spinal cord injury at home, let’s say you fell off your roof while putting up Christmas lights, then you would be under one of the long- term disability or extended health providers and they don’t provide case management. WSIB [Workplace Safety and Insurance Board] doesn’t provide that either. They would probably say that they case manage their own file but it’s very much in an entitlement system. It’s only the auto files that actually will purchase objective third party case managers but only for catastrophically impaired”*	Manager 8; Female Rehabilitation Manager
**Theme: Accessibility**	
**Representative quote**	**Source**
*“I know that there was a question of being able to…he always calls ahead kind of thing. If he knows certain exam rooms aren’t accessible, then we’ll try to request another one that’s easier for him to transfer. He definitely is aware of accessibility where he’s going to meet the doctors. I’m not sure if sometimes he might have to cram into a small room which is kind of ridiculous”*	Caregiver 4; Wife of individual with traumatic SCI
**Theme: Physical limitations and secondary complications**	
**Representative quote**	**Source**
*“I think she is frustrated. Let’s say she wants to do something and she cannot do it, let’s say opening the jar and she cannot open it because her hand is not that strong to open a jar. Then if she wants to get something, even though she has this picker, a device she uses in getting something…If she wants to do something, she uses that picker and then she’s having a hard time, that frustrates her. But there are only a few things that I know frustrates her, like putting the clothes on the hanger and then the jar opening. Those are the two things that I find are being frustrated”*	Caregiver 1; Personal support worker of individual with traumatic SCI
**Theme: Negative outlook or mood**	
**Representative quote**	**Source**
*“Then also that other thing I said where he doesn’t feel he deserves that stuff, he won’t advocate for himself because he’s just a guy in a wheelchair and he’s useless anyway right. That’s not a healthy attitude but I would say that that maybe something that comes up with other people where you know they may not advocate for themselves because they don’t think they’re worth it. It’s sad really”*	Caregiver 2; Wife of individual with traumatic SCI

#### Caregiver burnout

Caregiver burnout was identified as a major barrier to self-management on the part of individuals with traumatic SCI and this was well-recognized across all participant groups. Given the role that family members/caregivers play in care processes and overall well-being of individuals with traumatic SCI, several participants believed that caregiver burnout could threaten the sustainability of these critical supports. Indeed, the dual role of family members – most often wives in the current study – as both a spouse and performing the duties of a nurse was highlighted as a stressor. A lack of specialized or targeted services/programs for family members/caregivers to address this burden was also noted.

#### Funding and funding policies

Funding and funding policies that do not promote quality of life were also described across the participant groups as major barriers to self-management among individuals with traumatic SCI. In the absence of this funding and associated services and policies, family members are often required to fill these system-level gaps (e.g., provide attendant care in the absence of/as a result of decreased funding for homecare providers). Differences in the funding of services or different levels of service based solely on the mechanism of injury were also outlined.

#### Lack of accessibility

Accessibility was highlighted as another barrier to self-management across all of the participant groups. It was often discussed in terms of accessing buildings, and more specifically, difficulties with accessing physician offices and/or exam tables. One rehabilitation manager even suggested that as a result of these specific difficulties, individuals with SCI must access the emergency department to receive care.

#### Physical limitations and secondary complications

Physical limitations of the injury and secondary complications in and of themselves were recognized as barriers to self-management among individuals with traumatic SCI. These limitations may affect the ability to live well with a SCI and/or even the ability to carry out activities of daily living.

#### Difficulties achieving positive outlook or mood

Finally, a negative outlook or mood and/or lack of self-advocacy were identified as barriers to self-management, mirroring the identification of a positive outlook and acceptance as a facilitator to self-management. The impact of a concurrent traumatic brain injury was also described as a potential contributing factor to this negative outlook or mood or lack of self-advocacy, with some participants describing observable changes to personality and/or motivation/perseverance.

## Discussion

The current study aimed to understand the facilitators and barriers to self-management and is one of the few studies in the SCI literature to amalgamate the perspectives of individuals with traumatic SCI, their family members/caregivers, and acute care and rehabilitation managers. Using a descriptive qualitative approach, the five major facilitators were identified as: physical caregiver support, emotional caregiver support, peer support and feedback, positive outlook and acceptance, and maintaining independence/control over care. Conversely, the five major barriers were: caregiver burnout, funding and funding policies, accessibility, physical limitations and secondary complications, and negative outlook or mood or lack of self-advocacy. Collectively, and consistent with the Knowledge to Action framework [18] that guided the current study, these identified facilitators and barriers could inform implementation considerations for self-management programs for individuals with traumatic SCI and their caregivers (see Table 4 for Summary).

**Table 4:** Summary of identified facilitators and barriers to self-management, corresponding self-management program components, and implementation considerations in individuals with traumatic spinal cord injury and their caregivers

**Identified facilitators (F), barriers (B)**	**Suggested self-management program components/modules**	**Implementation considerations**
-Physical support from the caregiver (F)	-Caregiver component (i.e., skills training and emotional support)	-Training and support for caregivers needs to be responsive to the evolving needs of individuals with traumatic SCI (i.e., as they age and/or develop chronic conditions)
-Emotional support from the caregiver (F)		-The sustainability of caregiver activities and support required in SCI may be affected by aging and/or the chronic health conditions among caregivers themselves
-Caregiver burnout (B)		
-Peer support and feedback (F)	-Peer support component	-Matching peer mentors and mentees by specific demographic and clinical/injury characteristics (age, sex, etiology of injury) should be considered
		-Timing of support should be considered (e.g., acute phase of recovery may be too early)
-Maintaining Independence/Control over Care (F)	-Self-efficacy component	-Time since injury may play an important role in (increasing) self-efficacy in traumatic SCI
-Importance of Positive Outlook and Acceptance (F)	-Mood (depression) component (or Mind-Body component)	-Time since injury may play an important role in (increasing) mood in traumatic SCI
-Difficulty Achieving Positive Outlook or Mood (B)		-Physical limitations and secondary complications, chronic conditions, and co-morbid traumatic brain injury should be considered
-Physical Limitations and Secondary Complications (B)		
-Funding and Funding Policies (B)	-Awareness/knowledge of various funding programs	-Health system factors (funding, accessibility) need to be optimized for overall self-management optimization among individuals with traumatic SCI and their caregivers
-Lack of accessibility (B)	-Advocacy skills training	

### Role of caregivers

Collectively, this study demonstrated that caregivers, in this case, mainly wives of individuals with traumatic SCI, are making significant contributions to the physical and emotional well-being of individuals with traumatic SCI (i.e., their self-management). Indeed, it is well-recognized that the caregivers of individuals with SCI often become the primary source of help for a wide range of activities including bathing, dressing, and feeding the individual with SCI as well as providing bowel and bladder care [25], and as such, assume an *“unexpected career”*[26]. Despite the complexity of some of these tasks, it has been previously reported that caregivers of individuals with SCI may enter this new role without preparation or specialized training [27,28]. While many of the caregiver and manager participants acknowledged some skills training for caregivers at the rehabilitation phase of recovery, the need for ongoing training that is responsive to the evolving needs of individuals with traumatic SCI should be considered.

Furthermore, in performing these activities, the dual role of caregivers – as both a spouse and performing the duties of a nurse was highlighted as an important stressor. Dickson and colleagues [28] identified a related theme of *“post-injury shift in relationship dynamics”* (i.e., re-definition of the partner role) in their study on the impact of assuming the primary caregiver role following traumatic SCI. This identified change in relationship dynamics was associated with a loss of identity in the caregiver (i.e., either a complete loss of identity or the emergence of a new “caregiver” identity), highlighting the extent of the caregiver role in SCI, as does the current study. This role change from husband or wife to caregiver has also been found in stroke caregivers [29]. However, unlike stroke, the functionality of the individual with SCI does not improve over time, and thus, the activities/level of support of caregivers of individuals with SCI may not diminish over time [28].

While previous studies have emphasized the (physical) tasks associated with caregiving in SCI [25,28], the current study also highlighted the significant emotional support that caregivers provide. This emotional support may contribute directly to increased self-management to reduce secondary complications among individuals with traumatic SCI or act as an important contributor in a path of actions/behaviours that lead to increased self-management (e.g., the emotional support provided by the caregiver increases the self-efficacy of the individual with traumatic SCI which is in turn associated with increased self-management of secondary complications).

These physical and emotional contributions on the part of the caregivers of individuals with traumatic SCI are associated with a high level of caregiver burden, as identified in the current study and previous research. Indeed, elevated levels of physical stress, emotional stress, burnout, fatigue, anger and resentment, and depression among caregivers of individuals with SCI have been consistently reported [25,30-35]. Given the critical role of caregivers in self-management support of individuals with traumatic SCI, as well as the accompanying caregiver burden, interventions that address the ongoing needs of caregivers, including further skills training (e.g., as the individual with the traumatic SCI ages and has unique challenges brought about by the combined effects of aging and injury [36]) and psychological support are necessary. Two recent studies of interventions to assist family caregivers have demonstrated promise in reducing the impact of this burden [36,37], and in doing so, may promote ongoing care and/or sustainability of the care provided to individuals with traumatic SCI. Participants in these studies also raised the issue of aging and/or chronic health conditions among caregivers as a threat to the sustainability of caregiving activities, especially given the extent of support required in SCI.

### Role of peer support

Peer support also emerged as a significant facilitator to self-management among individuals with traumatic SCI. Learning from peers has been described as vital in the context of rehabilitation [38] and peer support, along with social support in general, has been shown to be a key factor in the adjustments for living with a SCI [39]. Haas and colleagues [40] determined that the main benefits of a community peer support service for individuals with SCI were the psychological and emotional support by a person with a SCI, advice on living with a SCI, practical advice and information, and ongoing support and friendship. These findings are consistent with those of the current study as well as the findings of other studies in SCI, which have reported the value of non-specific psychological and emotional support through the input of a peer mentor [39,41]. Furthermore, previous research has determined that practical advice provided by a peer mentor has the potential to add to the knowledge base of individuals with SCI, increase their self-efficacy, thereby increasing the likelihood of adherence to self-management behaviours to prevent secondary medical conditions [42]. The importance of matching peer mentors and mentees by specific demographic and clinical/injury characteristics was identified in the current study. The importance of this matching has been recognized in previous studies whereby demographic/clinical information such as age, race, and etiology of injury was considered when assigning a potential mentee to a mentor [42]. Lastly, the issue of appropriate timing for mentorship activities was raised, with some SCI participants recognizing that the acute phase of recovery may be too early to receive support.

### Role of (perceived) control and self-efficacy

Across all participant groups, the theme of maintaining control over care also emerged as a facilitator to self-management. Indeed, having a sense of control has consistently been found to have adaptive effects. In general, perceived control is associated with emotional well-being, reduced physiological impact of stressors, enhanced ability to cope with stress, improved performance, reduced pain, and a greater likelihood of making difficult behaviour changes [43]. Self-efficacy, defined as an individual’s belief or confidence in his or her capabilities to successfully execute the necessary courses of action to satisfy situational demands in the future, including those that are novel and stressful, is a construct of perceived control [44,45]. Decreased self-efficacy has been identified as a major factor to the lack of adherence with health and disease self-management [46], and thus it is not surprising that perceived control emerged as a facilitator to self-management in the current study. For example, it has been demonstrated that individuals with SCI who have higher self-efficacy demonstrate better mental health [47] and fewer secondary complications [48]. Pang and colleagues [15] demonstrated that those individuals with increased time since injury have better self-efficacy, but the results did not reach statistical significance. They concluded that it may take time for the newly injured individual to accept his or her own disabilities and learn to cope with the consequences of the condition and/or develop a better sense of control.

### Role of mood

The impact of mood was identified as both a facilitator and barrier to self-management in the current study. Indeed, the risk for major depression as well as anxiety disorder, post-traumatic stress disorder, substance abuse, and suicide is elevated for individuals with SCI compared with the general population [49-54]. It has been suggested that activating inherent psychological resources including skills, knowledge, experiences, and behavioural patterns, may protect individuals with SCI from the negative secondary consequences of the injury [55], supporting the current study’s findings. It has been previously concluded that depression can *“lead to apathy in terms of self-care”*[56]. However, studies also support the idea of *“feedback loops”* where certain patient factors and increased complications following depression may in turn accentuate the initial depression [57]. Indeed, secondary complications were identified as a barrier to self-management in the current study and thus may be acting as barriers in and of themselves (i.e., decreases in functional capacity) or may be contributing to this described feedback loop. The impact of traumatic brain injury (i.e., associated with the initial injury) on the mood and/or the ability to self-advocate, as well as the impact of other co-morbid conditions, such as diabetes and heart disease, in SCI and/or secondary conditions was raised. As was identified in the discussions around peer support and self-efficacy, it was acknowledged that the role of mood/self-advocacy on self-management behaviour is not static and may evolve as the length of time since injury increases.

### Role of accessibility and funding policies

Limited accessibility to services and/or equipment and in some cases an associated lack of funding or funding policies that did not support accessibility were identified as barriers to self-management. In support of this finding, Lund and colleagues [58] determined that individuals with SCI who perceive no serious barriers to participation in their daily lives report levels of life satisfaction that are similar to a healthy population. This suggests that perceptions of diminished quality of life following SCI might be a consequence of environmental barriers and inequity of opportunity rather than the SCI itself (i.e., impairment, secondary conditions) or the individual’s own personal characteristics [59,60]. Moreover, the negative impact of limited mobility, societal barriers, inadequate/inaccessible services and uninformed policies on the development of preventable complications has only been recently recognized, suggesting that inequitable community access may contribute to perceptions of poor quality of life and secondary conditions, and that these consequences are inter-related. Thus, while improvement of the individual-level factors discussed above (i.e., self-efficacy, mood) may lead to increased self-management of secondary complications, the current findings suggest that health system-level factors (e.g., access and availability of services, models of care) also need to be improved in order to create the optimal conditions for self-management among individuals with traumatic SCI [35].

### Limitations

The current study acknowledges some limitations. In terms of the recruitment procedure, it is likely that a selection bias operated in those participants who agreed to take part in the research – they may have been healthier and/or had better/more interest in self-management skills than those individuals who chose not to participate. Additionally, all participants had to have a caregiver who was willing to participate. At the same time, a certain level of intrinsic motivation and support is required in order to successfully participate in a self-management program [61-63], and thus it could be argued that the appropriate input was obtained for the development of a self-management intervention for individuals with traumatic SCI and their caregivers. The majority of traumatic SCI participants in the current study were male, which is consistent with the epidemiology of population-based studies [64], with female caregivers. However, future research should attempt to focus on the perspective of females with a traumatic SCI as well as the perspectives of male caregivers in order to increase the applicability of the study findings. Lastly, health care (or clinical) managers from adult acute care/trauma and rehabilitation centres were included in order to triangulate the findings from a health care professional and/or health system perspective. However, it is acknowledged that some managers are disconnected from the reality of practical day-to-day issues facing patients and their families, even the managers who were once clinicians before moving into management.

### Future research

Further research is required in the area of interventions to promote caregiver well-being, especially given caregivers’ critical role to the individual with traumatic SCI and the health care system as a whole. Further research is also required on the emotional support that caregivers provide to individuals with traumatic SCI and the associated patient outcomes. The issue of aging and/or chronic health conditions among caregivers as a threat to the sustainability of caregiving activities was also raised, especially given the extent of support required in SCI. Future research/interventions should address this important consideration. There is a need to further examine the mechanisms of the relationships and inter-relationships between mood (i.e., depression), self-efficacy, self-management adherence, and secondary complications in SCI. Future research could also determine a more comprehensive list of mentor/mentee characteristics and what specific characteristics or combination of characteristics are tied with increased self-management among individuals with traumatic SCI. Across several of the themes (peer support, perceived control and self-efficacy, mood), the issue of timing/readiness was raised. Again, future research should determine the appropriate levels of mentorship/support at varying stages of recovery in order to optimize moderating/mediating variables or outcomes of self-management (e.g., increased self-efficacy, decreased number of secondary conditions). The role of aging and comorbidity in SCI, in general, and as they relate to the identified themes warrant further research. Finally, current self-management programs [44,45] lack these components/considerations (e.g., emotional support for caregivers, issue of timing/readiness), and thus their relevance to generic and/or disease-specific programs could also be investigated for program development.

## Conclusions

The current study is, to the best of our knowledge, the only study on the facilitators and barriers to self-management in traumatic SCI, which captures the perspectives of the individual with traumatic SCI, their caregivers, and health care professionals. Overall, the current study demonstrated that the caregiver, peer support, perceived control and self-efficacy, mood, and accessibility and funding policies make significant contributions to the self-management of individuals with traumatic SCI. The issues of timing/readiness and comorbidities and aging were observed across many of these themes. As such, the development of a tailored self-management program for individuals with traumatic SCI and their caregivers should incorporate these considerations (i.e., see Table 4). It is anticipated that such a program could have a significant impact on reducing secondary complications, attenuating caregiver burnout, and enhancing quality of life for individuals with SCI.

## Abbreviations

SCI: Spinal cord injury

## Competing interests

The authors declare that they have no competing interests.

## Authors’ contributions

SEPM designed the study, in consultation with all other authors. SEPM collected the data and analyzed the data in consultation with FW and SBJ. All authors reviewed and revised the manuscript critically for important intellectual content. All authors read and approved the final manuscript.

## Pre-publication history

The pre-publication history for this paper can be accessed here:

http://www.biomedcentral.com/1471-2377/14/48/prepub

## Supplementary Material

Additional file 1: Interview Guides. Click here for file
