# Supplementary material for: Perceived facilitators and barriers to self-management in individuals with traumatic spinal cord injury: a qualitative descriptive study
Source: BMC Neurol. 2014 Mar 13;14:48. doi: 10.1186/1471-2377-14-48 (PMC4007626; doi:10.1186/1471-2377-14-48)
Supplement: Additional file 1: — Interview Guides. [file 1471-2377-14-48-S1.pdf]

Interview Guide for Individuals with Traumatic Spinal Cord Injury

A. Background Information

Date of Interview:

Time of Interview:

Thank the individual for participating, go over consent form and have him/her verbally agree to participate, explain process, how confidentiality and anonymity will be protected, etc. Underscore the fact that we are having a conversation; it is *his or her* experiences and input that are important. Underscore that they don't have to answer any questions that they are uncomfortable answering.

B. Warm-up and Establishing Rapport

- It would be nice if you could let me know a little about yourself [this information may have been obtained ahead of time]:
  - How the injury occurred (mechanism of injury) and when he or she sustained his or her injury
  - Level of injury
  - Age
  - Race/ethnicity
  - Marital status
  - Number of children
  - Living situation
  - Dwelling status i.e., house or apartment?
  - Rural/urban status
  - Education: less than high school, high school, undergraduate, postgraduate.

- Employment status
- Individual and family income: <$20,000, $20-40K, $40-60K, $60-80K, $80-100K, $100K+
- Insurance Status

C. Barriers and Facilitators of Self-Management

1. What are some of the factors that have contributed to success in self-management **(self-care activities which promote wellness or activities or strategies that you do to keep yourself healthy and/or manage or prevent secondary complications)?**

2. What are some of the factors that have impeded success in self-management **(self-care activities which promote wellness or activities or strategies that you do to keep yourself healthy and/or manage or prevent secondary complications)?**

D. Secondary Complications and Health Care Utilization

1. What are you currently doing to prevent any secondary complications**, that is, any medical conditions that arise as a result of your spinal cord injury, such as urinary tract infections or pressure ulcers?**

2. What prompted your last visit to hospital or your physician's office? Probes: What was that experience like? Were you comfortable talking to the physician about your health concerns? Did you feel as if you got the answers to all your questions and/or all of your concerns were *addressed?*

3. What kind of *specific* help did you need after that visit *(attendant care, etc*.)? Who or what helped you meet these needs? Was there anything that was not helpful?

E. Wrap-up Questions

- Is there anything else I haven't asked you about that you'd like to add?
- The responses you have provided may stimulate some additional questions or need for further clarification. If so, may we contact you in the future?

Interview Guide for Family Members/Caregivers/Attendants of Individuals with Traumatic Spinal Cord Injury

A. Background Information

Thank the individual for participating, go over consent form and have him/her verbally agree to participate, explain process, how confidentiality and anonymity will be protected, etc.

B. Warm-up and Establishing Rapport

It would be nice if you could let me know a little about yourself [including age, gender, relationship to individual with SCI].

C. Barriers and Facilitators of Self-Management

1a. What are some of the factors that have contributed to success in self-management (of the patient) **(self-care activities which promote wellness or activities or strategies that you do to keep yourself healthy and/or manage or prevent secondary complications)?**

1b. What are some of the factors that have contributed to success in *your own* self-management **(self-care activities which promote wellness or activities or strategies that you do to keep yourself healthy and/or manage or prevent secondary complications)?**

2a. What are some of the factors that have impeded success in self-management (of the patient) **(self-care activities which promote wellness or activities or strategies that you do to keep yourself healthy and/or manage or prevent secondary complications)?**

2b. What are some of the factors that have impeded success in *your own* self-management **(self-care activities which promote wellness or activities or strategies that you do to keep yourself healthy and/or manage or prevent secondary complications)?**

D. Secondary Complications and Health Care Utilization

1. What are you currently doing to help the patient [insert name] prevent any secondary complications**, that is, any medical conditions that arise as a result of your spinal cord injury, such as urinary tract infections or pressure ulcers?**

2. What prompted the patient's [insert patient's name] last visit to hospital physician's office?

Probes: What was that experience like? Was [insert patient's name] comfortable talking to the physician about his/her health concerns? Did he/she feel as if he/she got the answers to all his/her questions and/or all of the concerns were *addressed?* What kind of help did he/she need

after that visit? Who or what helped to meet these needs? Was there anything that was not helpful?

E. Wrap-up Questions

- Is there anything else I haven't asked you about that you'd like to add?
- The responses you have provided may stimulate some additional questions or need for further clarification. If so, may we contact you in the future?

Interview Guide for Rehabilitation and Acute Care Managers in Ontario

A. Background Information

Date of Interview:

Time of Interview:

Thank the individual for participating, go over the consent form with him/her and have him/her verbally agree to participate, explain process, how confidentiality and anonymity will be protected, etc. Underscore the fact that we are having a conversation; it is *his or her* experiences and input that are important. Underscore that they don't have to answer any questions that they are uncomfortable answering.

B. Warm-up and Establishing Rapport

- It would be nice if you could let me know a little about yourself [this information may have been obtained ahead of time]:
  - Position
  - Number of years in position
  - Age
  - Sex
  - Rehabilitation or acute Care?
  - Number of patients on unit/in program/number of beds

C. Barriers and Facilitators of Self-Management

1. What could be added to your program to assist patients with self-management support/skills? **Probes: Any barriers, any facilitators to making this addition at the individual, provider, and/or policy levels?**

2. What do you believe are the facilitators to self-management for patients in the community? **Probes: Again at the individual, provider, and/or policy levels?**

3. What do you believe are the barriers to self-management for patients in the community? **Probes: Again at the individual, provider, and/or policy levels?**

4. What could be added to your program to assist family members or caregivers with self-management support/skills? **Probes: Any barriers, any facilitators to making this addition at the individual, provider, and/or policy levels?**

5. In your opinion, what facilitates self-management for family members or caregivers in the community? **Probes: Again at the individual, provider, and/or policy levels?**

6. What do you believe are the barriers to self-management for family members or caregivers in the community? **Probes: Again at the individual, provider, and/or policy levels?**

D. Wrap-up Questions

- Is there anything else I haven't asked you about that you'd still like to add regarding self-management programs?
- The responses you have provided may stimulate some additional questions or need for further clarification. If so, may we contact you in the future?
